# The UDP-Glucuronate Decarboxylase Gene Family in *Populus*: Structure, Expression, and Association Genetics

**DOI:** 10.1371/journal.pone.0060880

**Published:** 2013-04-16

**Authors:** Qingzhang Du, Wei Pan, Jiaxing Tian, Bailian Li, Deqiang Zhang

**Affiliations:** 1 National Engineering Laboratory for Tree Breeding, College of Biological Sciences and Technology, Beijing Forestry University, Beijing, China; 2 Key Laboratory of Genetics and Breeding in Forest Trees and Ornamental Plants, Ministry of Education, College of Biological Sciences and Technology, Beijing Forestry University, Beijing, China; University of Umeå, Sweden

## Abstract

In woody crop plants, the oligosaccharide components of the cell wall are essential for important traits such as bioenergy content, growth, and structural wood properties. UDP-glucuronate decarboxylase (UXS) is a key enzyme in the synthesis of UDP-xylose for the formation of xylans during cell wall biosynthesis. Here, we isolated a multigene family of seven members (*PtUXS1-7*) encoding UXS from *Populus tomentosa*, the first investigation of UXSs in a tree species. Analysis of gene structure and phylogeny showed that the *PtUXS* family could be divided into three groups (*PtUXS1/4, PtUXS2/5, and PtUXS3/6/7*), consistent with the tissue-specific expression patterns of each *PtUXS*. We further evaluated the functional consequences of nucleotide polymorphisms in *PtUXS1*. In total, 243 single-nucleotide polymorphisms (SNPs) were identified, with a high frequency of SNPs (1/18 bp) and nucleotide diversity (π_T_ = 0.01033, θ_w = _0.01280). Linkage disequilibrium (LD) analysis showed that LD did not extend over the entire gene (*r* ^2^<0.1, *P*<0.001, within 700 bp). SNP- and haplotype-based association analysis showed that nine SNPs (*Q* <0.10) and 12 haplotypes (*P*<0.05) were significantly associated with growth and wood property traits in the association population (426 individuals), with 2.70% to 12.37% of the phenotypic variation explained. Four significant single-marker associations (*Q* <0.10) were validated in a linkage mapping population of 1200 individuals. Also, RNA transcript accumulation varies among genotypic classes of SNP10 was further confirmed in the association population. This is the first comprehensive study of the *UXS* gene family in woody plants, and lays the foundation for genetic improvements of wood properties and growth in trees using genetic engineering or marker-assisted breeding.

## Introduction

With the rapid increases in global industrialization, economic development, and human populations, the world faces potentially serious energy shortages and environmental problems [1]. Forests represent approximately 27% of the world’s land area, and wood is a major renewable resource for timber, paper and emerging bioenergy industries [2]. Therefore, a fundamental understanding of cellulose biosynthesis may enable us to enhance carbon sequestration and meet greater demands for biofuels [3]. Among forest trees, poplar is emerging as a model woody crop because it has several key advantages over other trees, including flexibility of harvest time, substantial carbon allocation to stems, rapid growth, high biomass, minimal requirements for cultivation and lower amounts of fermentation-inhibiting extractives, resulting in higher biomass conversion efficiency [3], [4]. Based on these natural characteristics and the substantial genetic diversity within *Populus*, the development of fast-growing, high-yield poplars, with improved wood quality has the potential to enable sustainable forest development, allowing both industrial and environmental improvements.

Wood (secondary xylem) is produce by cell division, cell expansion (elongation and radial enlargement), cell wall thickening (involving cellulose, hemicelluloses, cell wall proteins, and lignin biosynthesis and deposition), programmed cell death, and heart wood formation [5]. The secondary walls are composed of cellulose, lignin and hemicelluloses, including xylans and glucomannans. Cellulose and lignin provide mechanical strength to the secondary walls, and hemicelluloses form cross-links among cellulose microfibrils, which are thought to be important for cell wall assembly. In the wood of dicot species, xylan is the second most abundant polysaccharide after cellulose, and UDP-xylose (UDP-Xyl) is a nucleotide sugar required for xylan synthesis [6], [7]. In plants, the biosynthesis of UDP-Xyl is catalyzed by different membrane-bound and soluble UDP-glucuronic acid decarboxylase (UXS) isozymes, which irreversibly convert UDP-GlcA (UDP- glucuronic acid) to UDP-Xyl. Thus, UXS represents a key enzyme for partitioning glycosyl residues between the hexosyl and pentosyl residues. In addition, because of its central role in sugar nucleotide interconversion, UXS is likely ubiquitous among plants and a target for regulatory control during cell wall biosynthesis [8]. The first *UXS* gene was identified from *Cryptococcus neoformans* by bioinformatics methods [6]. Subsequently, *UXS* genes have been cloned from only a few plants; for example, the *Arabidopsis thaliana UXS* family contains six members grouped into three classes based on their genomic structure and subcellular localization [9]. Rice also has six *UXS* genes dispersed throughout the genome, and these were classified into three types by phylogenetic analysis [10]. In the Poaceae, such as barley (*Hordeum vulgare)*, analysis of transcript levels of the *UXS* members reveals that they likely have specific functions in cell wall formation during plant development [11]. In tobacco (*Nicotiana acum*), antisense downregulation of UDP-glucuronate decarboxylase leads to high glucose-to-xylose ratios in xylem walls due to fewer xylose-containing polymers. Such plants also have altered vascular organization and reduced xylans in their secondary walls [12]. Semiquantitative real time PCR analysis in cotton (*Gossypium hirsutum*) showed that *GhUXSs* transcripts were preferentially expressed during fiber development, from elongation through secondary cell wall synthesis [8]. These studies on non-woody species show that *UXS* family members are expressed throughout plant growth and development as they influence cell wall structure. Therefore, it is crucial to enhance our understanding of the role of *UXSs* in regulating growth and wood fiber properties in forest tree species.

The complex biological characteristics and long generation intervals of trees hinder the improvement of wood quality through conventional breeding methods. Given these constraints, traditional breeding of forest trees can be enhanced by marker-assisted selection (MAS), with advantages including reduced breeding cycle time, reduced cost of field testing, and increased efficiency and precision of selection [13], [14]. In this way, the selection of target traits can be achieved indirectly using molecular markers that are closely linked to underlying genes. Advances in high-throughput technologies for sequencing and genotyping and new genomic resources have enabled genome-wide examination of the number and effect of candidate genes related to traits of interest, through complex trait dissection using linkage disequilibrium (LD) mapping [15]–[18]. In recent years, SNP-based association genetics and LD mapping have enabled new MAS strategies in forest trees [19], [20]. In particular, candidate gene-based association approaches have been particularly useful to identify alleles associated with growth and wood properties in several tree species, such as conifers [21]–[25] and Eucalyptus [26]–[29]. In recent years, as the genome of *Populus trichocarpa* has been completely sequenced, poplar is increasingly considered as a model tree for genome-wide identification and characterization of gene families involved in growth and development [30]. For example, a set of candidate gene SNP associations was identified with chemical wood properties in *Populus trichocarpa* [31] and *Populus nigra* [32].

In this study, we used poplar as a model to first address the significance of *UXS* function and multiplicity in trees. We report the identification and characterization of the *UXS* gene family members, from the economically important tree *Populus tomentosa* [3], [33]. Transcript profiling revealed that the *UXS* genes may play important roles in wood formation. Furthermore, we used association tests to examine the allelic effects of natural variation in *PtUXS1* on growth and wood-property traits and validated a set of allelic effects by LD mapping to identify useful alleles located within functional genes controlling phenotypic traits.

## Results

### Isolation of Seven Distinct cDNAs Clones from *P.* *tomentosa*

We used reverse transcription (RT)-PCR to isolate seven full-length cDNAs from a cDNA library prepared from the mature xylem zone of *P*. *tomentosa*. The seven cDNA clones *PtUXS1-7* (GenBank Accession No. KC311162 - KC311168) were 1129 bp to 1800 bp in length, with open reading frames encoding polypeptides of 343 to 443 amino acid residues (Table 1), and 5′UTR and 3′UTR sequences that varied from 47 bp to 618 bp and 34 bp to 374 bp, respectively. Nucleotide sequences comparison of *PtUXS1-7* cDNAs with known full-length *Arabidopsis UXS* cDNA sequences showed that *PtUXS1* and *PtUXS4* were 69.8% and 68.4% identical to *AtUXS1*; *PtUXS2* and *PtUXS5* were 71.4% and 67.9% identical to *AtUXS2*; *PtUXS3*, *PtUXS6* and *PtUXS7* were 78.3%, 70.7% and 69.9% identical to *AtUXS5*. In addition, the corresponding estimated molecular masses and isoelectric points (pI) ranged from 38.5 kD to 49.7 kD and 6.73 to 9.42, respectively (Table 1).

**Table 1:** *UXS* gene family members in *Populus*.

Gene	cDNA (GenBank)	Genomics (GenBank)	cDNA length (bp)	Genomic DNAlength (bp)	Amino acids	kDa	pI
*PtUXS1*	KC311162	KC311169	1800	4374	430	48.3	8.94
*PtUXS2*	KC311163	KC311156	1466	3158	443	49.7	8.14
*PtUXS3*	KC311164	KC311157	1129	4397	343	38.5	8.43
*PtUXS4*	KC311165	KC311158	1451	4154	424	47.5	8.70
*PtUXS5*	KC311166	KC311159	1594	3153	439	49.4	9.42
*PtUXS6*	KC311167	KC311160	1249	3848	346	39.1	6.73
*PtUXS7*	KC311168	KC311161	1287	3313	346	39.0	7.66

PtUXSs contain all of the conserved features of the UXS family. For example, all PtUXS family members have several sequence motifs, including an N-terminal GxxGxxG sequence that is characteristic of an ADP-binding *βαβαβ*-fold associated with NAD (P)-binding proteins [9] (Figure S1). The seven PtUXSs can be classified into two groups. Class I includes PtUXS1, PtUXS2, PtUXS4 and PtUXS5, which have the same amino acid residues-GGAGFVG (Figure S1). Class II includes PtUXS3, PtUXS6 and PtUXS7, which also have the same amino acid residues-GGAGFIG (Figure S1). As reported for Arabidopsis, rice and cotton UXSs, the PtUXS family contains a characteristic and highly conserved Ser, Tyr, and Lys triad, of which, Lys and Tyr are in the YxxxK motif. Although the core catalytic domain of the PtUXS was conserved, variable regions were identified in the N and C termini. In addition, analysis of the seven PtUXSs using PSORT program (http://www.psort.org/) indicated that PtUXS1, PtUXS2, PtUXS4 and PtUXS5 have a transmembrane domain (at residues 49–65, 45–61, 46–62 and 45–61, respectively) in the N-terminal region (Figure S1).

### Genomic Organization of the *PtUXS* Family

**Figure 1:**
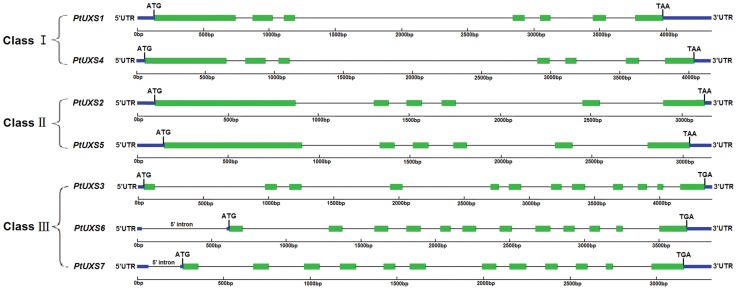
Genomic organization of *PtUXSs*. Three patterns of intron-exon structures of the *PtUXS* genes were identified and designated I, II and III. Exons and untranslated regions (UTRs) are shown as green and blue boxes, respectively, and the lines between boxes indicate introns.

To examine changes in intron/exon structure during evolution, we compared the full-length genomic sequences of the *PtUXS* family (GenBank Accession No. KC311169 and KC311156 - KC311161) and determined the intron/exon organization of each gene (Table 1 and Figure 1). The number (5 to 11) and length of introns (78 bp to 1797 bp) varied (Figure 1). All introns start with 5′G–T and end with 3′A–G among all the *PtUXS* family members and are in accordance with the GT–AG rule for a splicing site. Although strong conservation in the coding sequences and positions of exon/intron boundaries was detected in all *PtUXS* genes, the sizes and sequences of the introns among the seven *PtUXS* genes were found to be significantly divergent. Three patterns of intron-exon structures of the *PtUXS* genes were identified and designated I, II and III (Figure 1). Pattern I (*PtUXS1* and *PtUXS4*) includes a large intron 3 of 1656 bp and 1797 bp, and the other five small introns that vary from 88 to 327 bp and 95 to 361 bp in length, and 90.0% identity of the cDNA sequences (Table 2). Pattern II (*PtUXS2* and *PtUXS5*) includes two small introns (introns 2 and 3) and three medium introns (introns 1, 4, and 5), with lengths ranging from 100 to 700 bp and 103 to 487 bp; these two genes have a high cDNA sequence identity of 90.8% (Figure 1 and Table 2). Pattern III, which includes *PtUXS3*, *PtUXS6* and *PtUXS7,* had 11 introns in the encoding regions and the positions and lengths of them were similar (Figure 1). However, the structures of *PtUXS6* and *PtUXS7* had more identity, and contain small introns in the 5′UTR comprising 568 bp and 174 bp at −14 bp upstream of the ATG initiation codon (Figure 1).

**Table 2:** Coding region nucleotide (upper portion of matrix) and amino acid (bottom portion of matrix) sequence pairwise comparisons (% similarity) between *PtUXSs*.

	*PtUXS1*	*PtUXS2*	*PtUXS3*	*PtUXS4*	*PtUXS5*	*PtUXS6*	*PtUXS7*
*PtUXS1*	–	63.8	61.5	90.0	62.8	60.1	58.6
*PtUXS2*	70.3	–	60.5	66.0	90.8	62.3	59.3
*PtUXS3*	66.2	66.4	–	61.2	61.3	76.9	76.8
*PtUXS4*	92.7	69.9	66.2	–	63.8	57.4	57.2
*PtUXS5*	70.5	91.8	67.3	70.1	–	59.5	58.6
*PtUXS6*	66.8	66.1	90.1	66.5	67.3	–	84.5
*PtUXS7*	66.5	66.7	90.4	66.2	67.8	94.8	–

### Evolution of *UXS* Genes in Angiosperms

**Figure 2:**
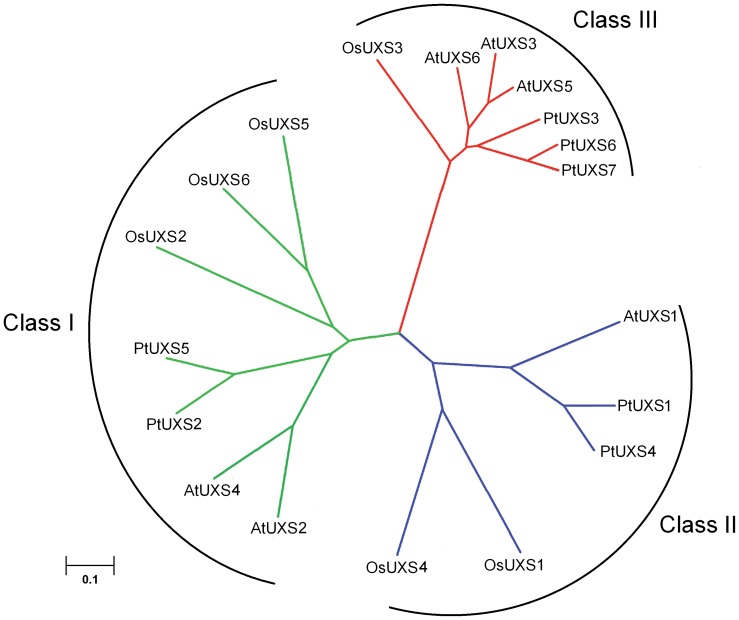
An unrooted phylogenetic tree of UXS members from poplar (PtUXS), *Arabidopsis* (AtUXS) and rice (OsUXS). Protein sequences of *Arabidopsis* UXS family members (AtUXSs) and rice UXS members (OsUXSs) were obtained from the (National Center for Biotechnology Information; http://blast.ncbi.nlm.nih.gov/Blast.cgi ).

To clarify the evolutionary relationship between the *PtUXS* genes and other angiosperm *UXS* genes, a neighbor-joining (NJ) tree was constructed with 19 complete amino acid sequences of UXS from *P*. *tomentosa*, *A*. *thaliana* and *Oryza sativa* (Figure 2). The phylogenetic dendrogram formed three well-defined subgroups (Classes I, II, and III), consistent with the intron-exon structure. Class I contained PtUXS2/5, AtUXS2/4 and OsUXS2/5/6, Class II consisted of PtUXS1/4, AtUXS1 and OsUXS1/4, and PtUXS3/6/7, AtUXS3/5/6 and OsUXS3 formed the third sub-group (Class III). The amino acid sequence similarity found between PtUXS1 and PtUXS4 was 92.7% and the similarity between PtUXS2 and PtUXS5 was 91.8% (Figure 2 and Table 2). Although PtUXS3, PtUXS6 and PtUXS7 were classified into the same sub-group, PtUXS6 and PtUXS7 were more closely related to each other than they were to PtUXS3. Also, their sequence similarity at the protein level was 94.8% (Figure 2 and Table 2). From the results of phylogenetic analysis, we inferred that the UXS family members split off before the species diverged.

### Transcript Profiling in Different Tissues and Organs

**Figure 3:**
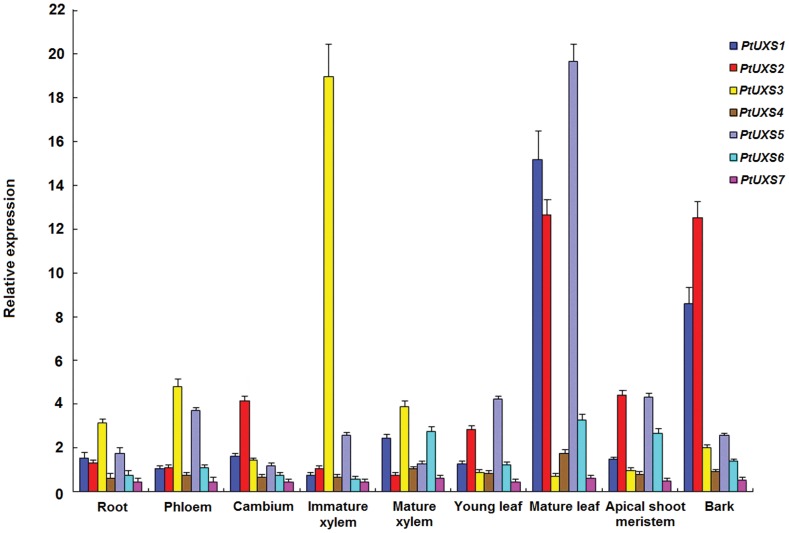
Relative transcript levels of seven *PtUXS* family members in different tissues and organs. Transcript levels were measured in different tissues, as indicated, by quantitative RT-PCR and is normalized to expression of *Actin*. Error bars represent+SD.

To determine the spatial expression patterns of the *PtUXS* members, real-time quantitative PCR was used to measure transcript abundance in different tissues and organs (Figure 3). *PtUXS* family members were differentially expressed in the tissues and organs tested and exhibited different expression patterns. All the family members except *PtUXS3* were most abundantly expressed in mature leaf, and *PtUXS3* had the highest expression levels in immature xylem of stem, but was lowest in mature leaf (Figure 3). *PtUXS1* and *PtUXS4* had similar expression patterns, and they were both moderately expressed in the mature xylem and bark (Figure 3). However, *PtUXS1* was expressed at higher levels than *PtUXS4* in all tissues; for example, expression of *PtUXS1* in mature leaf was almost eight-fold higher than that of *PtUXS4* (Figure 3). *PtUXS2* and *PtUXS5* were both expressed at the highest levels in the mature leaf and showed lower expression levels in the mature xylem (Figure 3). In addition, *PtUXS6* and *PtUXS7* were most abundantly expressed in the mature leaf, and they also had moderate expression levels in the mature xylem and apical shoot meristem, but showed the lowest expression in the immature xylem of stem (Figure 3). Therefore, the transcript profiles of these *PtUXS* genes appeared to be consistent with their genomic structure and phylogenetic relationships. Also all the *PtUXSs* appear to be involved in the development of various tissues and organs of poplar, but at different expression levels and with different tissue expression profiles.

### Nucleotide Diversity of *PtUXS1* in Natural Populations

To characterize the intraspecific molecular evolution of the poplar *UXS* genes, we first obtained genomic sequence of *PtUXS1* from 44 unrelated individuals in a discovery population that represents almost the entire natural range of *P*. *tomentosa*. An approximately 4374 bp genomic region of *PtUXS1*, including 133 bp of 5′UTR, 1293 bp of coding regions, 2574 bp of intron, and 374 bp of 3′UTR, was amplified and sequenced. Table 3 summarizes the statistical analysis of nucleotide polymorphisms (excluding indels) over different regions of *PtUXS1*. Across the samples, 243 SNPs were detected in *PtUXS1*, at a high frequency, 1/18 bp (Table 3). The SNP frequencies in the different gene regions were: 1/19 bp in the 5′UTR, 1/21 bp in exons, 1/17 bp in introns, and 1/15 bp in the 3′UTR. The lowest level of nucleotide polymorphism was found in the coding region, suggesting that the region is conserved relative to the other regions under selective pressure. In the coding sequence, 34 of the 62 SNPs located in the exons of *PtUXS1* led to nonsynonymous changes (including 32 missense and 2 nonsense mutations) to the amino acid sequence (Table 3). The other 28 SNPs produced no changes to the amino acid sequence, and were categorized as synonymous mutations. 209 SNPs were categorized as totally silent in the whole gene (Table 3). In total, 82 of the 243 SNPs (34%) were considered common (frequency >0.10). Generally speaking, the *PtUXS1* locus has high nucleotide diversity, where π_T_ = 0.01033 and θ_w = _0.01280 (Table 3). More specifically, estimates of nucleotide diversity (π_T_) for the different gene regions ranged from 0.00225 (exon 3) to 0.02391 (intron 4) with θ_w_ ranging from 0.00497 (Exon 6) to 0.02407 (Intron 4). Within coding regions, the value of non-synonymous nucleotide substitutions (π_nonsyn_) was markedly lower than π_syn_, with a π_nonsyn_/π_syn_ ratio of 0.17, suggesting that diversity at the non-synonymous sites of exon regions resulted from strong purifying selection (Table 3).

**Table 3:** Nucleotide polymorphism in *PtUXS1*.

Region	No. of bp	No. of polymorphic sites	Percentage polymorphism	Nucleotide diversity	
				π	θ_w_
5′UTR	133	7	5.26	0.01274	0.01055
Exon 1	609	29	4.76	0.01266	0.01105
Synonymous	143.83	13	9.04	0.03637	0.02226
Non-synonymous	456.17	16	3.51	0.00519	0.00752
Intron 1	135	5	3.70	0.00478	0.00520
Exon 2	145	5	3.45	0.00524	0.00789
Synonymous	33.33	2	6.00	0.01346	0.00686
Non-synonymous	110.67	3	2.71	0.00276	0.00827
Intron 2	88	7	7.95	0.02088	0.01840
Exon 3	80	3	3.75	0.00225	0.00880
Synonymous	15.22	1	6.57	0.00292	0.01501
Non-synonymous	59.78	2	3.35	0.00296	0.01148
Intron 3	1676	84	5.01	0.00893	0.01219
Exon 4	84	6	7.14	0.00714	0.01634
Synonymous	16.26	3	18.45	0.00651	0.03371
Non-synonymous	64.74	3	4.63	0.00735	0.01078
Intron 4	120	17	14.17	0.02391	0.02407
Exon 5	75	4	5.33	0.00294	0.01220
Synonymous	17.67	2	13.32	0.00743	0.02589
Non-synonymous	57.33	2	3.49	0.00155	0.00798
Intron 5	327	19	5.81	0.01224	0.01329
Exon 6	93	2	2.15	0.00305	0.00497
Synonymous	21.33	2	9.38	0.01316	0.02144
Non-synonymous	68.67	0	0	0	0
Intron 6	228	17	7.46	0.01462	0.01759
Exon 7	207	13	6.28	0.00319	0.01436
Synonymous	44.38	5	11.27	0.00687	0.02577
Non-synonymous	159.62	8	5.01	0.00223	0.01146
3′UTR	374	25	6.68	0.01484	0.01720
Total silent^a^	3269.78	209	8.93	0.01228	0.01420
Synonymous	295.78	28	9.47	0.02210	0.02088
Non-synonymous	976.22	34	3.48	0.00385	0.00820
Total *PtUXS1* b	4374	243	5.56	0.01033	0.01280

a Total silent = synonymous plus silent sites.

b Total *PtUXS1* = silent sites plus Non-synonymous sites.

Regions containing indels are excluded from the calculation.

### Linkage Disequilibrium

**Figure 4:**
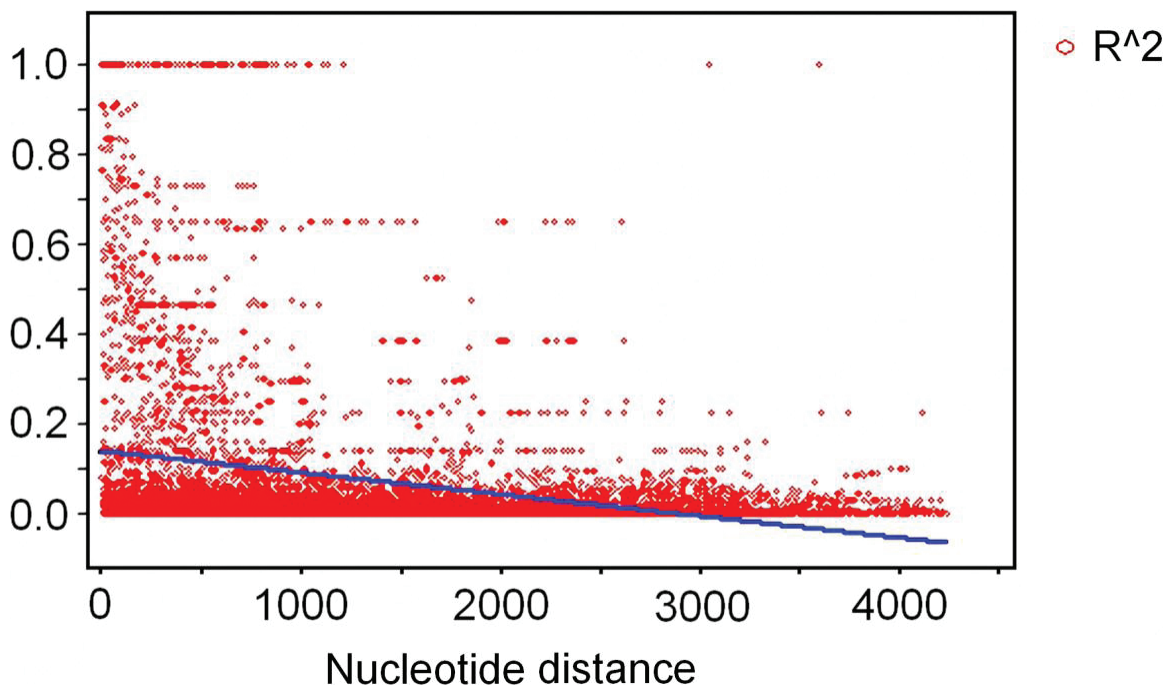
Decay of linkage disequilibrium within *PtUXS1* gene. Pairwise correlations between SNPs are plotted against the physical distance between the SNPs in base pairs. The straight line describes the least squares fit of *r* ^2^ (Er2) to its expectation. Linkage disequilibrium has largely decayed within 700 bp.

The decay of LD within *PtUXS1* was shown by a plot of *r* ^2^ against distance in base pairs between SNPs (Figure 4). In the *P*. *tomentosa* population, the level of LD decayed rapidly, with *r* ^2^ values declining to 0.1 within 700 bp, indicating that LD did not extend over the entire gene region. The low LD observed in this study suggested that the resolution of associations between the marker and trait will be high. Using genotype data of 82 SNPs from 426 individuals in the association population, the analysis of LD displayed six high-LD distinct haplotype blocks within *PtUXS1* (r^2^>0.75), including SNP 4–7, SNP 9–16, SNP 18–20, SNP 26–28, SNP 31–34 and SNP 62–64 (Figure S2).

### Single Marker-trait and Haplotype-based Associations

Single-marker associations between 82 SNPs and 10 growth and wood quality traits were conducted using the mixed linear model (MLM). In total, 25 significant associations representing 16 SNP loci were identified at the threshold of *P*<0.05 (Table S1). However, correction for multiple testing using the FDR method resulted in only 9 significant associations (*Q* <0.10, Table 4). Table 2 lists highly significant associations identified with seven traits, including holocellulose content, α-cellulose content, fiber length, fiber width, microfibril angle, the diameter at breast height (D) and stem volume (V). These markers explained a small proportion of the phenotypic variance, with individual effects ranging from 2.70% to 12.37% (Table 4). Of these markers, SNP2 from 5′UTR and SNP22 from intron 2 both showed significant association with holocellulose content (Table 4). The non-synonymous marker SNP6 in exon 1, which results in an encoded amino acid change from Tyr to His, associated significantly with multiple traits, i.e., fiber width, V and D. Also, SNP10 in exon 1, a synonymous mutation, associated with α-cellulose content (Table 4). Of the remaining noncoding markers, SNP27 and SNP56 were both closely associated with fiber length, and SNP27 explained the highest proportion of the phenotypic variance (12.37%); also, SNP 68 was significantly associated with microfibril angle (Table 4). We calculated the gene actions for each significant marker-trait association. One of the nine marker-trait associations showed evidence of overdominance (|d/a| >1.25), and the remaining eight associations were split between modes of gene action that were codominant (|d/a| ≤0.5, 2), and partially to fully dominant (0.50< |d/a| <1.25, 6) (Table 5).

**Table 4:** Summary of significant SNP marker-trait pairs from the association test results in the discovery (association population) and validation (linkage mapping population) populations after a correction for multiple testing errors.

Trait	Locus	Position	Association population (*N* = 426)			Linkage mapping population (*N* = 1200)		
			*P-*value	*Q*-value	*R* ^2^ (%)	*P-*value	*Q*-value	*R* ^2^ (%)
Holocellulose	SNP 2	5′UTR	0.0015	0.0229	3.86	0.0051	0.0402	4.03
	SNP 22	Intron 2	0.0073	0.0492	2.80			
α-cellulose	SNP 10	Exon 1	0.0042	0.0356	3.85	0.0104	0.0716	3.98
Fiber length	SNP 27	Intron 3	2.33E−10	5.592e−08	12.37			
	SNP 56	Intron 4	2.79E−04	0.0107	4.64	0.0310	Q >0.10	3.50
Fiber width	SNP 6	Exon 1	0.0017	0.0272	2.70	0.011	0.0720	5.63
Microfibril angle	SNP 68	Intron 6	0.0138	0.0762	2.77			
Breast height diameter (D)	SNP 6	Exon 1	3.12E−08	2.496e−06	9.67	0.009	0.0590	3.07
Stem volume(V)	SNP 6	Exon 1	2.89E−08	2.496e−06	9.72			

*P*-value = the significant level for association (the significance is *P*≤0.05), *R^2^* = percentage of the phenotypic variance explained, *Q*-value = a correction for multiple testing [false discovery rate FDR (*Q*) ≤0.10].

**Table 5:** List of marker effects for significant marker–trait pairs in the discovery population.

Trait	SNP	2a^1^	d^2^	d/a	2a/sp^3^	Frequency^4^	a^5^
Holocellulose	SNP2	1.1154	0.0619	0.1109	0.1037	0.47(C)	−3.5109
	SNP22	1.7470	3.2853	3.7608	0.1624	0.49(T)	−1.7705
α-cellulose	SNP10	1.3871	0.3247	0.4682	0.1562	0.49(C)	4.8354
Fiber length	SNP27	0.0758	0.0240	0.6316	0.9028	0.48(A)	−0.0288
	SNP56	0.0172	0.0095	1.1024	0.2049	0.44(C)	0.0023
Fiber width	SNP6	1.7748	0.9877	1.1131	0.8954	0.47(T)	−0.01120
Microfiberangle	SNP68	1.6288	0.8427	1.0347	0.3600	0.48(C)	−1.2067
D	SNP6	1.7632	1.0698	1.2135	0.3146	0.47(T)	0.5013
V	SNP6	0.0715	−0.0228	−0.6376	0.1780	0.47(T)	−0.0151

D* = *the diameter at breast height, V = stem volume.

1 Calculated as the difference between the phenotypic means observed within each homozygous class (2a = |G_BB−_G_bb_|, where G_ij_ is the trait mean in the ijth genotypic class).

2 Calculated as the difference between the phenotypic mean observed within the heterozygous class and the average phenotypic mean across both homozygous classes [d = G_Bb_−0.5(G_BB_+G_bb_), where G_ij_ is the trait mean in the ijth genotypic class].

3 s_p_, standard deviation for the phenotypic trait under consideration.

4 Allele frequency of either the derived or minor allele. Single nucleotide polymorphism (SNP) alleles corresponding to the frequency listed are given in parentheses.

5 The additive effect was calculated as a = p_B_(G_BB_)+p_b_(G_Bb_)-G, where G is the overall trait mean, G_ij_ is the trait mean in the ijth genotypic class and p_i_ is the frequency of the ith marker allele. These values were always calculated with respect to the minor allele.

Using the haplotype trend regression method, 12 common haplotypes (frequency >1%) were found to be significantly associated with growth and wood-quality traits (Table 6). Of these, one haplotype from SNP 1–3 and two haplotypes from SNP 21–23 showed genetic associations with holocellulose content; two haplotypes from SNP 27–29 and one haplotype from SNP 56–58 were associated with fiber length; two haplotypes were associated with fiber width, and one haplotype each with α-cellulose content, microfibril angle, D and V traits were observed in the association population (Table 6). The proportion of phenotypic variation explained by these haplotypes varied from 3.00% to 8.82%, and eight single-marker associations (*Q* <0.05), strongly supporting the haplotype-based associations for these traits (les 4 and 6).

**Table 6:** Haplotypes significantly associated with growth and wood property traits.

Trait	*P*-value	*R^2^*(%)	Significant haplotypes	Frequency	Single-marker associations
Holocellulose	0.1702	4.55	SNPs 1–3		SNP 2 (Holocellulose, *Q = *0.0229)
			A-C-G	0.3565	
	0.0258	4.61	SNPs 21–23		SNP 22 (Holocellulose, *Q = *0.0492)
			T-G-G	0.0536	
			A-T-G	0.1735	
α-cellulose	0.0243	5.08	SNPs 10–12		SNP 10 (α-cellulose, *Q = *0.0356)
			G-T-A	0.1263	
Fiber length	0.0214	8.82	SNPs 27–29		SNP 27 (Fiber length, *Q = *5.592e−08)
			A-A-C	0.038	
			G-G-T	0.0755	
	0.2032	5.26	SNPs 56–58		SNP 56 (Fiber length, *Q = *0.0107)
			G-C-G	0.3016	
Fiber width	0.0302	3.00	SNPs 4–6		SNP 6 (Fiber width, *Q = *0.0272)
			T-C-T	0.1279	
	0.0413	3.51	SNPs 23–25		/
			G-G-T	0.0239	
MFA	0.0344	4.05	SNPs 68–70		SNP 68 (MFA, *Q = *0.0762)
			T-C-A	0.0239	
D	0.0039	3.73	SNPs 32–34		/
			C-T-G	0.489	
V	0.0121	8.24	SNPs 5–7		SNP6 (V, *Q = *2.496e−06)
			C-T-T	0.426	

MFA* = *microfiber angle, D* = *the diameter at breast height, V = stem volume; *P-*value = the significant level for haplotype-based association (the significance is *P*≤0.05); *R^2^* = percentage of the phenotypic variance explained. Single-marker associations with the lowest *Q* value (FDR *Q* ≤0.10) relating to the significant haplotype–trait association.

### Confirmation of Association Studies in a Linkage Mapping Population

**Figure 5:**
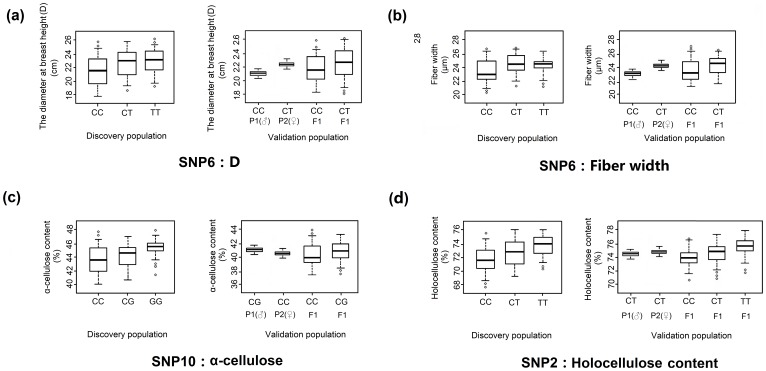
Genotypic effect on the significant single nucleotide polymorphisms (SNPs) found in *PtUXS1* with the same phenotypic trait in discovery and validation populations. (a–b) The nonsynonymous marker (SNP6) in exon1 of *PtUXS1* significantly associated with fiber width and D in discovery and validation populations, and shows patterns of gene action consistent with dominant effects. The T allele at SNP6 causes a Tyr to His amino acid substitution. (c) SNP10 in exon 1, a synonymous mutation, was associated with α-cellulose content in both populations, illustrating the pattern of gene action consistent with additive effects. (d) SNP2 from the 5′UTR of *PtUXS1* showed significant association with holocellulose content in both populations. The differences in holocellulose content among the three genotypes of this marker indicate that patterns of gene action are consistent with additive effects.

All 16 significant SNP markers (*P*<0.05; Table S1) identified in the discovery population were present in accordance with Mendelian expectations (*P*≥0.01), and no novel allele was discovered in the validation population. Therefore, single-marker association analysis (160; 16 SNPs ×10 traits) was conducted in the validation population. We first observed five marker-trait associations (*P*<0.05; Table 4), and subsequent multiple testing correction of *P*-values reduced the list of significant associations to four (*Q* <0.10; Table 4), with the percentages of phenotypic variation explained ranging from 3.07% to 5.63%. In the validation population, markers SNP2 and SNP10 were significantly associated with holocellulose and α-cellulose content, respectively, and explained 4.03% and 3.98% of the phenotypic variation (Table 4). In both the fiber width and D traits, the same significant marker SNP6 was observed, and they explained 5.63% and 3.07% of the phenotypic variance (Table 4). The mean phenotypic values among different genotypes in the four SNP markers showed significant differences, and the allelic effect of each marker was consistent in both association and validation populations (Figure 5).

### Transcript Analysis of SNP Genotypes

**Figure 6:**
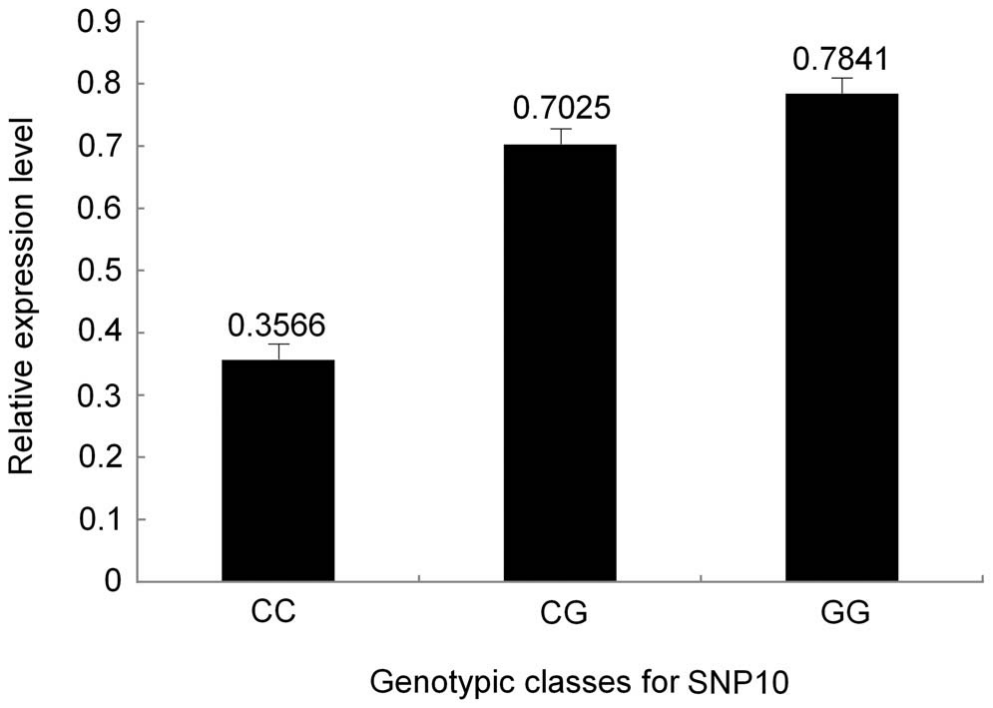
Expression levels of three genotypic classes for SNP10. The error bars represent+SD.

To determine whether these significant allelic SNPs affect the *PtUXS1* RNA transcript accumulation, transcript levels were compared among the different genotypic classes for seven significant SNPs (*Q* <0.10, Table 4) identified in association population using RT–qPCR with gene-specific primers. Measurement of differential expression across three or two genotypic classes (10 trees for each genotype) for each of the seven SNPs, indicated that SNP10 exhibited significant differences in the RNA transcript levels among the three genotypes in the association population (Figure 6). For the marker SNP10 (exonic), the highest relative expression levels of mRNA products were found in the GG group (0.7841), followed by the CG group (0.7025), and the transcript levels of the CC group were lowest (0.3566).

## Discussion

### Structure and Evolution of the *UXS* Family in *Populus*

Members of the *UXS* gene family have been found (based on EST daases) in monocots and dicots. However, the *UXS* gene family members cloned in poplar in our study are the first identified in a forest tree species. The *UXS* gene family is not restricted to higher plants because it was also identified from green alga (*Chlamydomonas reinhardtii*), human (*Homo sapiens*), rat (*Rattus norvegicus*), *Drosophila melanogaster*, and bacterial genomic daases [9], indicating that UXS proteins are evolutionarily conserved. In this study, we conducted a thorough analysis of the structure and evolution of the *UXS* family in the model tree *Populus* (Figure 1 and 2). The gene structure of the family is similar to *UXS* gene families reported in other plants [34]. The *UXS* genes share similarity with dehydratases, dehydrogenases, and epimerases. They all contain GxxGxxG NAD^+^ binding motifs and conserved Ser, Tyr and Lys amino acid residues that are believed to be located in the catalytic site. Previous reports on UDP-GlcA decarboxylase activities indicated that the subcellular localization of the enzymes from different sources varied, with some UDP-GlcA-DC isoforms cytosolic, and other isoforms membrane-bound [9]. Three of the six Arabidopsis *UXS* isoforms (AtUXS3, 5, 6) are predicted to be cytosolic (based on their sequence similarity to AtUXS3) and the other three (AtUXS1, 2, 4) are likely to reside in the endomembrane system (based on their similarity to AtUXS2) [35]. On this basis, we inferred that PtUXS1, PtUXS2, PtUXS4 and PtUXS5 reside in the endomembrane system, and the other three isoforms are cytosolic. Extensive sequence conservation across a broad range of plant taxa suggests that the UXS protein may have an essential function in growth and development in plant.

Analysis of the occurrence of *UXS* family members in complete genomes contributes to our knowledge of the origin and evolution of the plant UDP-glucuronate decarboxylase. In this study, we analyzed the evolution of the *UXS* family by classifying the family members of *A*. *thaliana*, *O*. *sativa* and *P*. *tomentosa.* This phylogenetic analysis shows the UXS family may split off before the species diverged; suggesting that all plant UXS family may have originated from ancestral types. Within the later gene duplication of UXSs within classes I, II, and III, this event occurred after the divergence of monocots and dicots. And then, the third subclass appears to be the recently split after the divergence of the woody plants (*Populus*) and the herbaceous (*Arabidopsis*).

In the *PtUXS* gene family, *PtUXS6* and *PtUXS7* have a 5′UTR intron of 568 bp and 174 bp, respectively (Figure 1). 5′UTR introns located close to the initiating ATG codon are thought to play an important role in gene expression from transcription to translation [36], [37]. For example, the rice *rubi3* promoter with 5′UTR intron conferred approximately 20-fold higher *GUS* expression than an intron-less version in transient assays in bombarded rice suspension cells [38]. In *Arabiopsis EF1α-A3*, the presence of a 5′UTR intron affects gene expression and the size of the 5′UTR intron influences the level of gene expression [39]. Thus, we speculated that the 5′UTR intron in *PtUXS6/7* may influence gene expression and regulation for the synthesis of UDP-xylose in *Populus*. The gene structure of the *PtUXS* family provides an important beginning to enable future exploration of the mechanisms of the evolution of gene function for each member and may help genetic engineers to regulate growth and development in trees for sustainable production of wood biomass in the future.

### *UXS* Families are Differentially Expressed in *Populus*

The significant divergence in the genomic structure of the poplar *UXS* genes, including the 5′UTR structure, suggested that these genes may differ in their expression levels or functions. In this study, the next step to understanding the respective functions of the poplar *UXS* family members was investigating their expression profiles. They appeared to be expressed throughout all stages of plant growth and development. Meanwhile, the tissue-specific expression pattern of each *UXS* family member provides a platform for understanding the functional roles of putative orthologs from different species. Among them, *PtUXS3* was predominantly expressed in the immature xylem of stem, and the other members, except *PtUXS2* and *PtUXS5*, also had moderate transcript levels in the mature xylem (Figure 3), suggesting that *UXSs* may be associated with wood cell wall biosynthesis. Previous studies showed that UXS catalyzes an irreversible reaction from UDP-GlcA to UDP-Xyl, which is subsequently converted to UDP-Ara by UDP-Xyl epimerase. Thus, UXS plays a central role in producing these pentose sugars in higher plants [10], [11]. Dalessandro and Northcote [40] studied the activity of the UXS enzyme in secondary cell wall synthesis in trees, and found that the activity of UXS enzyme improved substantially at the beginning of the stage at which the cambium formed immature xylem, whereas the activity decreased after the mature xylem formed. Wheatley et al [41] detected the activity of UXS in the cambium and differentiating vascular tissue of tobacco by immuno-hybridization. In barley, comparisons of transcriptional activities of the genes in various barley tissues showed that *HvUXS1* mRNA was relatively abundant in stems and the maturation zone of roots [11].

Moreover, *PtUXS1*, *PtUXS2* and *PtUXS4-7* all had the highest transcript levels in the mature leaf (Figure 3), suggesting that they were related to secondary meolites in synthesizing the cell wall. A similar phenomenon was also reported in the GT1 (glycosyltransferase 1) family of *P*. *trichocarpa*, which likely results from the fact that plants rely on enzymes to assimilate the products of photosynthesis into sugars and starch, synthesize cell wall biopolymers, and create various glycosylated compounds [42]. Similarly,the *CIP7* gene identified in seedlings and adult leaves of *A*. *thaliana* has also been shown to contribute to photosynthesis in the woody tissues or leaves of *Corymbia citriodora* subsp. *variegata*, and fixes CO_2_ to maintain stem internal CO_2_ produced by respiration and also contributes to plant growth [43]–[46]. Therefore, cell wall biosynthesis is coordinated with several other biological processes, and the *PtUXS* genes in these shared pathways often are functional homologs but from a different phylogenetic division [47]. Comparing the gene structure and tissue specific expression of *PtUXS* family members showed that their expression profiles are congruent with the evolutionary relationships based on protein sequences. These findings suggest that the most closely related *UXS* genes have similar expression patterns, whereas the more distant sub-groups have less similar patterns (Figure 1, 2 and 3). Thus, systematic tissue- and organ-specific expression studies of each *UXS* member are still needed to obtain a complete overview for the entire family.

### Linkage Disequilibrium Tests and Detection of Associations in *P. tomentosa*

LD-based association mapping plays an important role in increasing the resolution of marker-trait associations compared with traditional linkage mapping. Tree species are ideal for association mapping as they are predominantly outcrossing, have long recombination histories, and have large, effective, relatively unstructured populations, resulting in high levels of nucleotide diversity and low LD [19], [48]. Understanding the patterns of LD in the species is an important prerequisite for association mapping, because choosing genome-wide or candidate-gene-based associations depends on the patterns of LD decay in the species. In this study, LD declined rapidly within 700 bp in *PtUXS1* (*R* ^2^<0.1, *P*<0.001, Figure 4), which is consistent with the results of limited LD for candidate genes in other tree species, such as loblolly pine (*Pinus taeda* L) [22], [49], Scots Pine (*Pinus sylvestris*) [50], Douglas fir [51], and *Eucalyptus nitens* [26], [27]. In *Populus*, a rapid decay of LD occurs within just 300–1,700 bp in candidate genes among related species of *Populus*, based on SNP markers [16], [20], [31]. Therefore, candidate-gene-based LD mapping seems to be particularly useful in marker-assisted selection (MAS) breeding programs for trees. However, Slavov *et al* [52] found a slow decay of LD in the *P. trichocarpa* genome-wide level, with *r^2^* dropping below 0.2 within 3–6 kb, suggesting that genome-wide association studies may be more feasible in *Populus* than previously assumed. It should be noted that this study was not specifically designed to address LD in the genome, but rather within these specific genes. Further study of LD decay on a genome-wide level in trees remains to be conducted.

In this study, comparison of single-marker and haplotype-based associations (Table 4 and 6), demonstrated that the effect of the haplotype is mainly derived from an individual significant marker, suggesting that haplotype analysis may not be more powerful than single marker analysis in this low LD tree species. UXS enzymes play essential roles in the synthesis of hemicelluloses, glycoproteins and oligosaccharides. They are related to fiber formation and cross-linking polysaccharides that are synthesized during fiber elongation and secondary wall formation in plants [12], [53]. In wood, the hemicelluloses account for about 25% of the dry weight, and this implies that the *UXS* genes may have associations with wood fiber traits, of which little is known in tree species. This study identified significant associations between markers within candidate genes and growth and wood fiber traits.

Holocellulose is a combination of cellulose (a glucan polymer) and hemicellulose (mixtures of polysaccharides), accounting for nearly 80% of secondary xylem tissue and affecting mechanical strength [1], [5]. In this study, SNP2, located in the 5′UTR of *PtUXS1*, had a significant association (*Q* <0.10) with holocellulose content, with the same effects of genotype in both discovery and validation populations (table 4 and Figure 5). Also, the patterns of gene action are consistent with additive gene effects (Table 5). This finding suggests that SNP2 was a true positive in the linkage population, and it may be a functional polymorphism that controls holocellulose content. SNPs in 5′UTRs could affect phenotypic traits because 5′UTRs play crucial roles in the regulation of gene expression, especially at the transcriptional level [54], [55]. Sequences in the 5′ flanking region can affect mRNA sility, translational efficiency, or subcellular localization [56], [57]. SNP markers in the 5′UTR that significantly affect phenotypic traits in association studies have also been reported elsewhere. For example, Miyamoto et al [58] detected a significant SNP association in the 5′UTR of *GDF5* with hip osteoarthritis in two independent Japanese populations. Guerra et al [32] conducted association genetic studies of chemical wood properties in black poplar (*P*. *nigra*) and found that two highly significant SNP markers from the 5′UTR of *TUB15*, which encodes a β-tubulin, were associated with lignin content**.** The *UXS* gene family has been studied extensively in *Arabidopsis*, *Oryza* and *Gossypium*. For example, *AtUXS1*, the *Arabidopsis* ortholog of *PtUXS1*, encodes a UDP-GlcA decarboxylase, which converts UDP-GlcA to UDP-Xyl, and thereby regulates the synthesis of hemicellulose [59], [60]. In addition, *GhUXSs* are preferentially expressed during secondary cell wall synthesis [61]–[63] and antisense downregulation of *UXSs* may alter vascular organization and reduce xylans in cotton secondary cell walls [8].

For associations with α-cellulose content, we identified the marker SNP10, which is a synonymous mutation in exon 1 of *PtUXS1*, and found that its mode of gene action was consistent with additive effects (Table 5 and Figure 5). Since it is commonly believed that association studies with candidate genes should be preferentially conducted with functional SNPs, the identification of nucleotide substitutions associated with functional changes should have important implications for the design and interpretation of related association studies [64]. This conjecture was supported by the significant expression differences among three genotype classes of SNP10 in association population (Figure 6). Thumma et al [27] discovered a synonymous exonic SNP of *EniCOBL4A* associated with cellulose content and kraft pulp yield. Dillon et al [25] found a synonymous SNP in the second exon of an actin family member (*ACT7*), which was associated with cellulosic pulp yield. In addition, Kien et al [65] found that SNPs that affect the function or amount of actin may affect the amount or distribution of cellulose in the cell wall. In this study, although SNP10 is a synonymous variant positioned within the exon, it does not overlap with known regulatory motifs at the DNA sequence level or functional domains of the translated protein. Hence, the detailed functional effect of the marker in this gene must be further tested via other molecular approaches.

Our discovery that a non-synonymous exonic SNP (SNP6) in exon 1 of *PtUXS1* was significantly associated with both fiber width and diameter at breast height (D) may represent pleiotropic effects of *PtUXS1* [66]. A similar pleiotropic phenomenon has been identified in previous studies [17], [23]. In the discovery population, the differences in fiber width for the SNP6 marker were significant among three genotypes (Figure 5) and demonstrated a model of gene action consistent with dominant effects (Table 5). The T allele is the minor allele of this non-synonymous marker, and it represented a missense mutation that causes a Tyr→His amino acid substitution. The genotypic effects of SNP6 on diameter at breast height (D) were similar to that of association with fiber width (Figure 5). The results strongly suggest that SNP6 may be a functional polymorphism involved in the control of both fiber width and D. A similar study in maize identified a nonsynonymous SNP in the first exon of *C4H1* associated with forage quality traits [67]. The association between SNP6 and fiber width was consistent with findings that GhUXS is a key enzyme in determining the quality and integrity of cotton fibers, which are generated during a longer period of cellulose synthesis [68], and the association with D demonstrated that UXSs can accelerate the growth and development of plants. Undesirable negative correlations between wood quality and growth were not observed (data not shown), indicating the potential to break negative correlations by selecting for individual SNPs in breeding programs [24], [69].

In conclusion, in combination with previous reports, these association results indicate that *PtUXS1* affects fiber formation, growth and development, and wood quality of *P. tomentosa*. Hence, *PtUXS1* is an important candidate gene for future tree-breeding programs. In addition, associations with several SNP markers were detected in the linkage population. This validation has become the gold standard for assessing statistical results from association studies with large numbers of independent tests [70]. The validation can assist in ‘ruling in’ associations, and provided additional evidence for SNP effects [25]. In this case, the SNP markers identified in both discovery and validation populations of this study can be applied to breeding programs to improve the quality and quantity of wood products.

## Materials and Methods

### Plant Materials and Phenotypic Data

Discovery population: In 1982, 1047 native individuals collected from the entire natural distribution region of *P*. *tomentosa* were used to eslish a clonal arboretum, using a randomized complete block design with three replications, at Guan Xian County of Shandong Province from root segments [33]. In this study, the association population (discovery population) consisted of 426 unrelated individuals representing almost the whole geographic distribution of *P. tomentosa* (180 from the Southern region, 86 from the Northwestern region, and 160 from the Northeastern region) were used for the initial SNP association analysis. In addition, a panel of 44 unrelated individuals (15 from the southern region, 15 from the northwestern region, and 14 from the northeastern region) was sequenced to identify SNPs within *PtUXS1*.

Validation population: In this study, to confirm the association results using LD mapping, a validation population consisted of 1200 hybrid individuals were randomly selected from 5,000 F_1_ progeny eslished by controlled crossing between two elite poplar parents, clone “YX01” (*P*. *alba* × *P. glandulosa*) as the female and clone “LM 50” (*P. tomentosa*) as the male; these two species are members of the section *Populus*. The progeny were grown in 2008 in the Xiao Tangshan horticultural fields of Beijing Forestry University, Beijing, China (40°2′N, 115°50′E) using a randomized complete block design with three replications.

This study was carried out in strict accordance with the recommendations in the Guide for Observational and field studies. All necessary permits were obtained for the described field studies. The sampling of all individuals of *P*. *tomentosa* was approved by the Youhui Zhang, director of National Garden of *P*. *tomentosa*.

Phenotypic data: For all individuals in these two populations, ten traits were measured using the methods described previously [71], including lignin content, holocellulose content, alpha-cellulose content, fiber length, fiber width, microfibril angle, tree height (H), tree diameter at breast height (D), stem volume (V) and tree height/tree diameter (H/D). Analysis of variance (ANOVA) and phenotypic correlations for these ten traits in these two populations have been reported by Du et al [71] and Tian et al [69].

### Isolation of *PtUXS* cDNAs

The *P*. *tomentosa* stem mature xylem cDNA library was constructed using the Superscript λ System (Life Technologies). The cDNA library was generated as part of our large-scale effort to identify genes expressed predominantly in the mature xylem of *P*. *tomentosa* stems. The constructed cDNA library consisted of 5.0×10^6^ pfu with an insert size of 1.0–4.0 kb. Random end-sequencing of 10,000 cDNA clones and comparison with all available *Arabidopsis UXS* sequences revealed that seven EST sequences were highly similar to *AtUXSs*. Then, BLAST analysis of the seven EST sequences at JGI Daase (http://genome.jgi-psf.org/Poptr1/Poptr1.home.html) was used to detect seven full-length cDNAs of *UXS* from *P*. *trichocarpa*. Gene specific primers were designed based on the full-length cDNAs of *P*. *trichocarpa*; finally, seven full-length *UXS* cDNAs were identified from *P. tomentosa* and named *PtUXS1-7* cDNAs.

### DNA Extraction and Identification of *UXS* Genomic DNA

Total genomic DNA was extracted from fresh young leaves of each *P*. *tomentosa* individual using the Plant DNeasy kit (Qiagen China, Shanghai), following the manufacturer’s protocol. The primer sets used for the amplification of *UXS*s were designed based on the sequenced cDNAs of *PtUXS1-7*. PCR was performed in a final reaction volume of 25 µl containing 20 ng genomic DNA, 0.8 U *Taq* DNA polymerase (Promega), 50 ng forward primer, 50 ng reverse primer, 1× PCR buffer (Promega), and 0.2 mM each dNTP (Promega). PCR conditions were as follows: 96°C for 5 min, and 30 cycles of 95°C denaturation for 30 s, 56°C annealing for 30 s, and 72°C extension for 1 min, with a final extension at 72°C for 5 min. The PCR products were finally separated by capillary electrophoresis using an ABI3730×l DNA Analyzer (Applied Biosystems, Carlsbad, CA, USA), after confirmation of PCR amplification on a 1.5% agarose gel. The analysis of polymorphic loci was performed with GeneMapper v4.0 software (Applied Biosystems) using the LIZ 600 size standard (Applied Biosystems).

### RNA Extraction, cDNA Synthesis, and Tissue-specific Expression Analysis of *PtUXSs*

For RNA extraction, fresh tissue samples of root, leaf, and apex were collected from 1-year-old *P. tomentosa* clone “LM 50”. The wood-forming tissues of upright stems, including, developing and mature xylem tissues, were collected by scraping the thin (approximately 1.0 mm) and the deep layer on the exposed xylem surface at breast height; The other wood forming tissues including phloem and cambium, were collected as described [72]. All tissues were immediately frozen in liquid nitrogen and stored at –80°C.

Total RNA was extracted from various tissues using the Plant Qiagen RNAeasy kit (Qiagen China, Shanghai) according to the manufacturer’s instructions. Additional on-column DNase digestions were performed three times during the RNA purification using the RNase-Free DNase Set (Qiagen). RNA was then quantified and reverse transcribed into cDNA using the SuperScript First-Strand Synthesis system and the supplied polythymine primers (Invitrogen) [33].

Real-time quantitative PCR was performed on a DNA Engine Opticon 2 machine (MJ Research) using the LightCycler-FastStar DNA master SYBR Green I kit (Roche). The *PtUXSs*-specific and internal control (*Actin*) primer pairs (Table S2) were designed using Primer Express 3.0 software (Applied Biosystems). The PCR program included an initial denaturation at 94°C for 5 min, and 40 cycles of 30 s at 94°C, 30 s at 58°C, and 30 s at 72°C, and a final melt-curve of 70–95°C. The specificity of the amplified fragments was checked by the melting curve. All reactions were carried out in triplicate, and the data were analyzed using the Opticon Monitor Analysis Software 3.1 tool.

### Phylogenetic Analysis

To analyze the phylogenetic relationships between *PtUXSs* and the *UXS* genes from other species, the amino acid sequences of *UXS* family members, including those from dicotyledons and monocotyledons, were identified by searching public daases available at NCBI (http://www.ncbi.nlm.nih.gov). Phylogenetic and molecular evolutionary analyses were conducted using MEGA version 4.0, and the neighbor-joining method was used to build phylogenetic trees [73]. Bootstrap analysis was performed using 1,000 replicates.

### SNP Discovery and Genotyping

Of the seven *UXS* genes identified in the *P.tomentosa*, we selected *PtUXS1* to explore the pattern of nucleotide diversity and conduct candidate-gene-based association mapping analysis. In order to identify SNPs within the *PtUXS1,* the entire gene was sequenced and analyzed in 44 unrelated individuals from the association population, without considering Insertions/deletions (INDELs), using the software MEGA 4.0 and DnaSP4.90.1 [74]. All 44 sequences described have been deposited in the GenBank daases (GenBank Accession No. KC311169 - KC311212). Subsequently, common SNPs (minor allele frequencies >0.10) were genotyped by the single-nucleotide primer extension method using a Beckman Coulter sequencing system across all DNA samples.

### Data Analysis

Linkage disequilibrium analysis: To assess the pattern of linkage disequilibrium in the sequenced candidate gene region, the decay of LD with physical distance (base pairs) between SNP sites within *PtUXS1* was estimated by linear regression analysis of linkage disequilibrium in DnaSP program version 4.90.1. The squared correlation of allele frequencies *r* ^2^ [75] was used to test the LD between pairs of SNP markers using the software package HAPLOVIEW (http://www.broad.mit.edu/mpg/haploview.html). The interval value of the parameter varies from 0 to 1. The significance (*P*-values) of *r^2^* for each SNP locus was calculated using 100,000 permutations.

Association testing: In the association population (discovery population), all trait-SNP association tests between 82 SNP markers and 10 traits were conducted, using the mixed linear model (MLM) with 10^4^ permutations in the software package TASSEL Ver. 2.0.1 (http://www.maizegenetics.net/) [76]. The MLM can be described as follows: *y* = *µ* +*Qv+ Zu*+*e,* where *y* is a vector of phenotype observation, *µ* is a vector of intercepts; *v* is a vector of population effects; *u* is a vector of random polygene background effects; *e* is a vector of random experimental errors; *Q* is a matrix defining the population structure from STRUCTURE, and *Z* is a matrix relating *y* to *u*. Var (*u*) = G = *σ^2^_a_K* with *σ^2^_a_* as the unknown additive genetic variance and *K* as the kinship matrix (Yu *et al*., 2006). In this Q+K model, the relative kinship matrix (K) was obtained using the method proposed by Ritland [77], which is built into the program SPAGeDi, Ver. 1.2 [78], and the population structure matrix (*Q*) was identified based on the significant subpopulations (*K = *11) [79], as assessed according to the statistical model described by Evanno et al [80], using 20 neutral genomic SSR markers. The positive false discovery rate (FDR) method was applied to correct for multiple testing by using QVALUE software [81].

A panel of 16 SNPs (*P*<0.05, Table S1) producing significant associations in the discovery population using the MLM was genotyped in the validation population. Inheritance tests of all significant SNP loci were first examined in the validation population by performing a chi-squared (*χ* ^2^) test at the 0.01 probability level; and then SNP markers following Mendelian expectations (*P*≥0.01), were used in single-marker analysis in this hybrid population (excluding the genotype data involving null allele in each locus). Significant SNP loci detection was calculated by fitting the data to the model *y* = *µ*+*m_i_*+*e_ij_*, where *y* is the trait value, *µ* is the mean, *m_i_* is the genotype of the *i*th marker, and *e_ij_* is the residual associated with the *j*th individual in the *i*th genotypic class. The FDR method was used to correct for multiple testing.

Haplotype analysis: Haplotype frequencies from genotype data were estimated and haplotype association tests were done on a three-marker sliding window, using haplotype trend regression software [82]. The significances of the haplotype associations were based on 1000 permutation tests.

Modes of gene action: The modes of gene action were quantified using the ratio of dominant (d) to additive (a) effects estimated from least-square means for each genotypic class. Partial or complete dominance was defined as values in the range 0.50< |d/a| <1.25, whereas additive effects were defined as values in the range |d/a| ≤0.5. Values of |d/a| >1.25 were equated with under- or overdominance. Details of the algorithm and formulas for calculating gene action were previously described [17], [31].

## Supporting Information

Figure S1 **Comparison of the amino acid sequences of plant UXS enzymes.** Amino acid sequences of UXSs from *Populus* (PtUXS1-PtUXS7), cotton (GhUXS1, accession no. ACI46983.1), Arabidopsis (AtUXS1, accession no. AT3G53520.1), and rice (accession no. LOC_Os05g29990.1) were aligned using the DNAMAN 6.0 software. The conserved motifs GxxGxxG (NAD^+^-binding), YxxxK, and the transmembrane domain are highlighted in red.
(TIF)

Figure S2 **(a–f) Significant pairwise linkage disequilibrium (*r^2^*>0.75, *P*<0.001) between SNP markers.** The significant common genotyped SNP blocks 1–6 are shown on a schematic of *PtUXS1* and the pairwise r^2^ values are shown by color coding in the matrix below.
(TIF)

Table S1 **Summary of significant SNP marker-trait pairs identified at the threshold of** ***P*** **<0.05, using the mixed linear model (MLM) in the discovery population.**
(DOC)

Table S2 **The real-time PCR primers used in this study.**
(DOC)
