# Supplementary figures and images for: The UDP-Glucuronate Decarboxylase Gene Family in Populus: Structure, Expression, and Association Genetics
Source: PLoS One. 2013 Apr 16;8(4):e60880. doi: 10.1371/journal.pone.0060880 (PMC3629030; doi:10.1371/journal.pone.0060880)

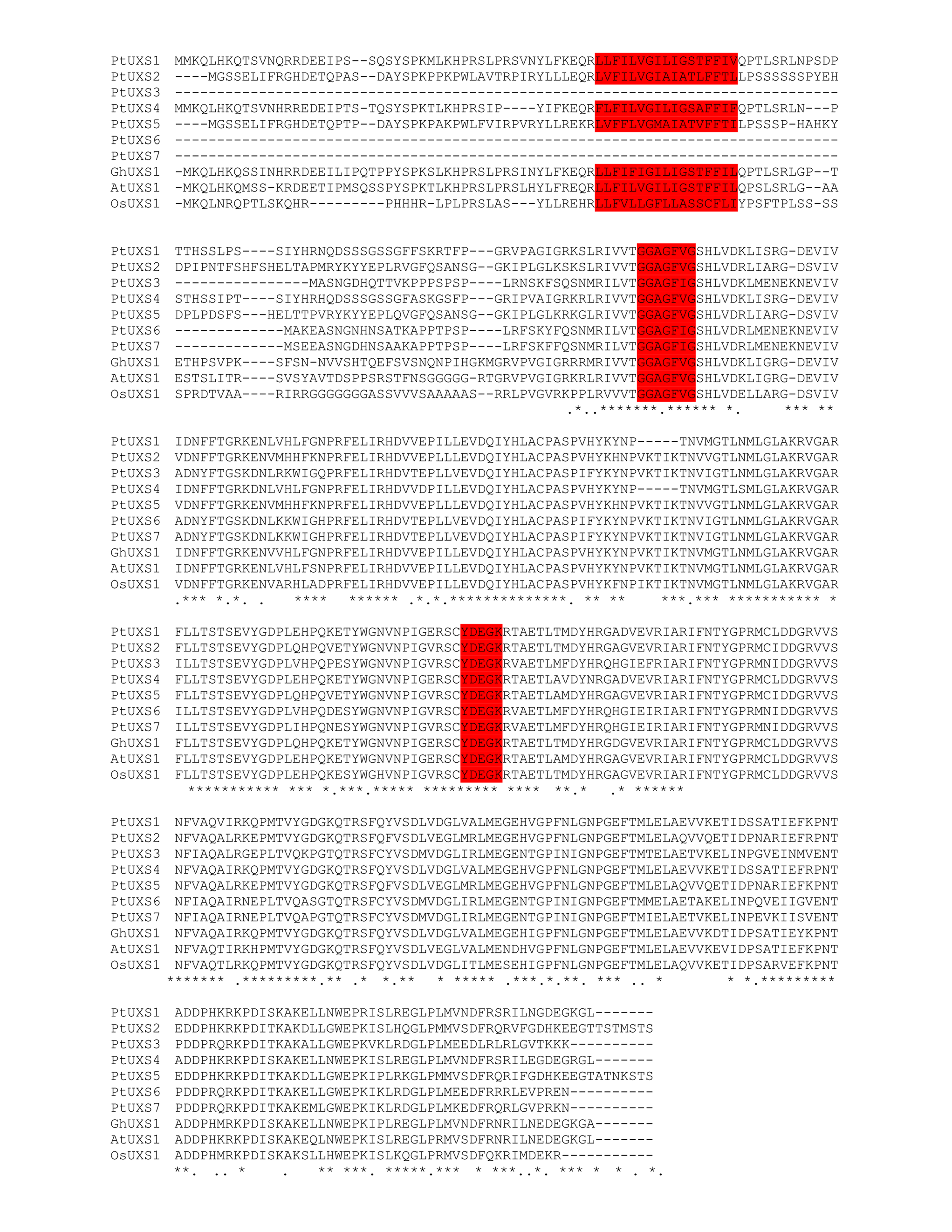

Supplement: Figure S1 — Comparison of the amino acid sequences of plant UXS enzymes. Amino acid sequences of UXSs from Populus (PtUXS1-PtUXS7), cotton (GhUXS1, accession no. ACI46983.1), Arabidopsis (AtUXS1, accession no. AT3G53520.1), and rice (accession no. LOC_Os05g29990.1) were aligned using the DNAMAN 6.0 software. The conserved motifs GxxGxxG (NAD+-binding), YxxxK, and the transmembrane domain are highlighted in red. (TIF) [file pone.0060880.s001.tif]

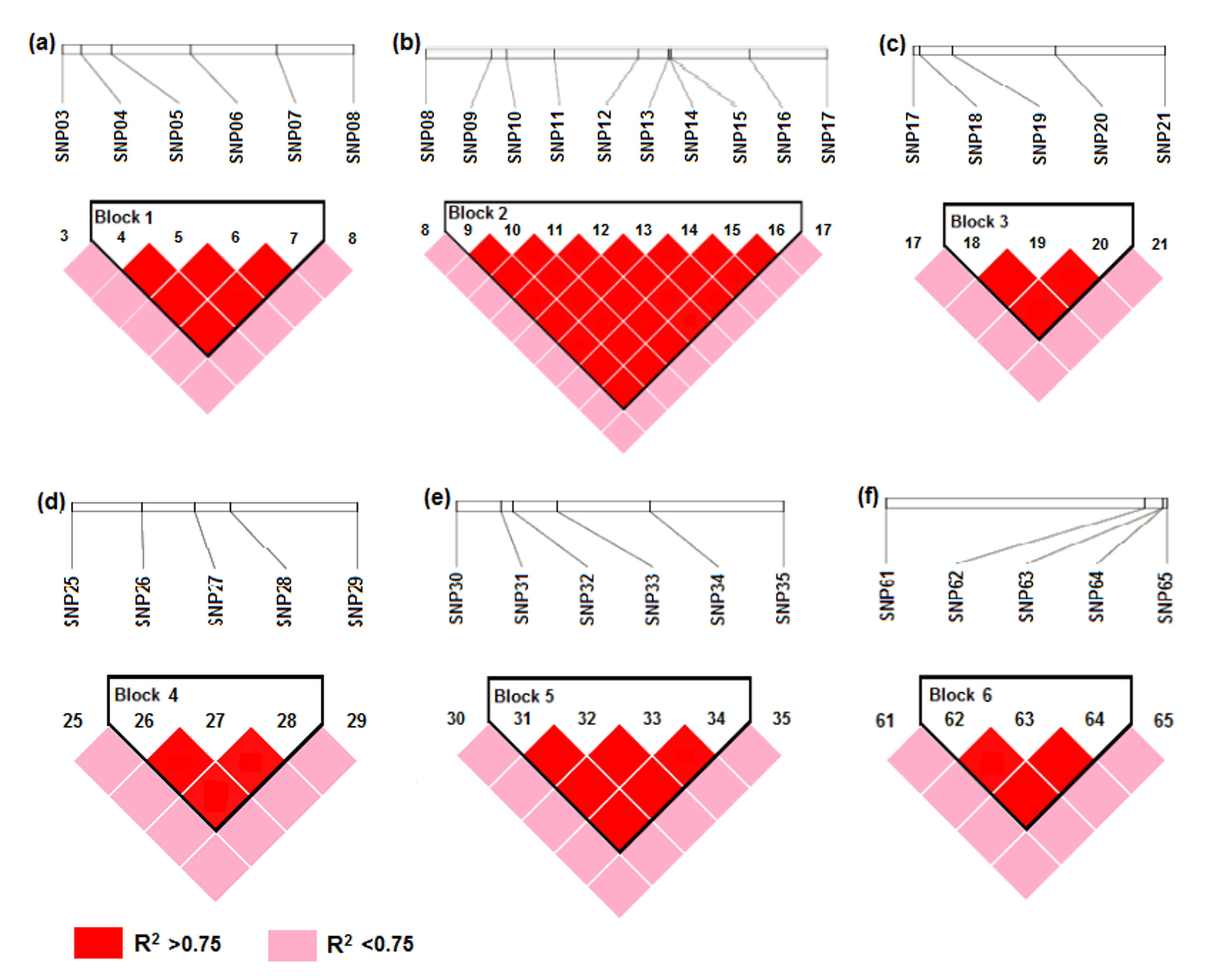

Supplement: Figure S2 — (a–f) Significant pairwise linkage disequilibrium (r2>0.75, P<0.001) between SNP markers. The significant common genotyped SNP blocks 1–6 are shown on a schematic of PtUXS1 and the pairwise r2 values are shown by color coding in the matrix below. (TIF) [file pone.0060880.s002.tif]
