# Supplementary material for: The UDP-Glucuronate Decarboxylase Gene Family in Populus: Structure, Expression, and Association Genetics
Source: PLoS One. 2013 Apr 16;8(4):e60880. doi: 10.1371/journal.pone.0060880 (PMC3629030; doi:10.1371/journal.pone.0060880)
Supplement: Table S1 — Summary of significant SNP marker-trait pairs identified at the threshold of P <0.05, using the mixed linear model (MLM) in the discovery population. (DOC) [file pone.0060880.s003.doc]

**Table S1**. Summary of significant SNP marker-trait pairs identified at the threshold of *P* < 0.05, using the mixed linear model (MLM) in the discovery population.

| Trait | Locus | Position | Association population ( *N* = 426) | | |
| --- | --- | --- | --- | --- | --- |
|  |  |  | *P-*value | *Q*-value | *R*^2^ (%) |
| Lignose | SNP21 | Intron 2 | 0.0368 | >0.10 | 2.08 |
|  | SNP30 | Intron 3 | 0.0283 | >0.10 | 2.24 |
| Holocellulose | SNP 2 | 5’UTR | 0.0015 | 0.0229 | 3.86 |
|  | SNP 22 | Intron 2 | 0.0073 | 0.0492 | 2.80 |
|  | SNP47 | Intron 3 | 0.0492 | >0.10 | 1.81 |
|  | SNP60 | Intron 4 | 0.0229 | >0.10 | 2.26 |
|  | SNP61 | Intron 5 | 0.0159 | >0.10 | 2.48 |
| α-cellulose | SNP2 | 5’UTR | 0.0215 | >0.10 | 2.21 |
|  | SNP 10 | Exon 1 | 0.0042 | 0.0356 | 3.85 |
|  | SNP22 | Intron 2 | 0.0486 | >0.10 | 1.74 |
|  | SNP42 | Intron 4 | 0.0231 | >0.10 | 2.17 |
| Fiber length | SNP6 | Exon 1 | 0.0267 | >0.10 | 1.26 |
|  | SNP22 | Intron 2 | 0.0305 | >0.10 | 1.79 |
|  | SNP 27 | Intron 3 | 2.33E-10 | 5.592e-08 | 12.37 |
|  | SNP53 | Intron 4 | 0.0241 | >0.10 | 1.91 |
|  | SNP 56 | Intron 4 | 2.79E-04 | 0.0107 | 4.64 |
|  | SNP64 | Intron 5 | 0.0166 | >0.10 | 2.10 |
| Fiber width | SNP 6 | Exon 1 | 0.0017 | 0.0272 | 2.70 |
|  | SNP60 | Intron 4 | 0.0258 | >0.10 | 2.00 |
| Microfibril angle | SNP10 | Exon 1 | 0.0437 | >0.10 | 1.67 |
|  | SNP27 | Intron 3 | 0.0382 | >0.10 | 1.74 |
|  | SNP 68 | Intron 6 | 0.0138 | 0.0762 | 2.77 |
| Breast height diameter (D) | SNP 6 | Exon 1 | 3.12E-08 | 2.496e-06 | 9.67 |
| Stem volume(V) | SNP 6 | Exon 1 | 2.89E-08 | 2.496e-06 | 9.72 |
| H/D | SNP14 | Exon 1 | 0.0345 | >0.10 | 1.10 |

*R^2^* = percentage of the phenotypic variance explained; *P*-value = significance level for association (significance is *P* ≤ 0.05); *Q*-value = a correction for multiple testing (false discovery rate FDR (*Q*) ≤ 0.10).
