# Supplementary material for: The UDP-Glucuronate Decarboxylase Gene Family in Populus: Structure, Expression, and Association Genetics
Source: PLoS One. 2013 Apr 16;8(4):e60880. doi: 10.1371/journal.pone.0060880 (PMC3629030; doi:10.1371/journal.pone.0060880)
Supplement: Table S2 — The real-time PCR primers used in this study. (DOC) [file pone.0060880.s004.doc]

**Table S2.** The real-time PCR primers used in this study

| Primer name | Primer sequences (5′-3′) | Size (bp) | *Ta* (°C) |
| --- | --- | --- | --- |
| *PtUXS1*RT | F: GGCTCTCTTCCGTAATCCATGTT  R: ACACGTACATCTTCCAGCTGAAGT | 72 | 58.0 |
| *PtUXS2*RT | F: CGTAGCAGCAACAACAGGAGAT  R: GCCAAAAGAACAGCTCCAAATC | 66 | 58.0 |
| *PtUXS3*RT | F: CCATTGCATCTGCCCTAGTTC  R: GGGTGAAGGCCATGCTCAT | 68 | 58.0 |
| *PtUXS4*RT | F: AATCCATGTTTGCGTGGAAATT  R: CCAAAAGAACAACTCCGAACCT | 84 | 58.0 |
| *PtUXS5*RT | F: GCGCAGCTTCCATCAAGAAT  R: TGTCACGTCCAAACCATTTATCTG | 68 | 58.0 |
| *PtUXS6*RT | F: AGTGAATTTGTCCGCAGCAGTT  R: TCCCAGGAGCAAGGAAACC | 77 | 58.0 |
| *PtUXS7*RT | F:TCTTTGCCTTCTGGGTGATTG  R:TTTGCCTCCCCATTAGACCTT | 58 | 58.0 |
| *Actin* | F:CTCCATCATGAAATGCGATG  R:TTGGGGCTAGTGCTGAGATT | 62 | 58.0 |

*Ta =* the optimized annealing temperature.
